# *Guium nebulum* gen. et sp. nov., a New Cup Moth from Southern China Based on Morphological and Molecular Analysis (Lepidoptera: Zygaenoidea: Limacodidae)

**DOI:** 10.3390/insects16010041

**Published:** 2025-01-03

**Authors:** Jun Wu, Ting-Ting Zhao, Hui Geng, Guang-Ze Jin, Hui-Lin Han

**Affiliations:** 1 School of Forestry, Northeast Forestry University, Harbin 150040, China; wujun5911@foxmail.com (J.W.); zhaotingting11@foxmail.com (T.-T.Z.); 2 Northeast Asia Biodiversity Research Center, Northeast Forestry University, Harbin 150040, China; taxus@126.com; 3 College of Life Science, Shangrao Normal University, Shangrao 334001, China; genghui1988iove@163.com; 4 School of Ecology, Northeast Forestry University, Harbin 150040, China; 5 Key Laboratory of Sustainable Forest Ecosystem Management, Ministry of Education, Northeast Forestry University, Harbin 150040, China

**Keywords:** DNA barcodes, molecular phylogeny, morphology, new genus, slug caterpillar moth, taxonomy

## Abstract

**Simple Summary:**

Limacodidae, known for its high species diversity in tropical and subtropical regions, includes over 300 genera and 1700 species globally, with China hosting a significant number of these. In this study, we describe a new genus and species within the family Limacodidae from Guangxi and Jiangxi, China. The new genus and species, *Guium nebulum* gen. et sp. nov., displays unique morphological features, including distinct wing patterns and male genital structures that differentiate it from related genera. Through an analysis of the COI mitochondrial gene, we further confirmed the genus’s monophyly.

**Abstract:**

A new genus and species of Limacodidae, *Guium nebulum* gen. et sp. nov., is described based on specimens collected from Guangxi Autonomous Region and Jiangxi Province in China. The new genus shares certain morphological features, such as a well-developed labial palpus, with related genera like *Tanvia Solovyev & Witt*, 2009; *Scopelodes* Westwood, 1841; *Hyphorma* Walker, 1865; and *Monema* Walker, 1855. However, the new genus can be separated from them by the wing venation and the male genital characteristics. COI molecular marker analysis further supports the monophyly of this new genus, indicating a close relationship with *Scopelodes*.

## 1. Introduction

The family Limacodidae, within the superfamily Zygaenoidea, is one of the largest families in this group, predominantly found in tropical and subtropical regions, with a high diversity in the Afrotropical and Oriental regions. The family comprises over 300 genera and 1700 species worldwide (van Nieukerken et al. [1]). Of these, China is home to 72 genera, 264 species, and 5 subspecies, representing over 15% of the known species, as noted by Wu (2010) [2] and by Wu and Fang (2023) [3]. Phylogenetic studies on this family have been relatively limited in the past. However, Liang et al. (2024) [4] recently reconstructed a higher-level taxonomic framework for Palaearctic and Indomalayan Limacodidae, recognizing six major clades within this family.

Guangxi Autonomous Region and Jiangxi Province are both located in southern China, with favorable natural conditions and high biodiversity. Wu and Fang (2023) [3] reported 79 species of the family in Guangxi, with a tie with Sichuan Province for the second-highest species richness in China, accounting for roughly one-third of the national total. The highest species diversity was reported in Yunnan Province, with 138 species. In comparison, Jiangxi reported 62 species, slightly fewer than Guangxi and Sichuan. During the field surveys in Guangxi and Jiangxi, an unknown Limacodidae species with elongated labial palpus was identified. Based on morphological and molecular analyses, we confirmed this species is a new species belonging to an undescribed genus.

Research on Limacodidae groups with elongated labial palpus can be traced back to Hering (1931) [5], who documented that several genera, including *Scopelodes* Westwood, 1841; *Monema* Walker, 1855; *Elassoptila* Turner, 1902; *Susica* Walker, 1855 (female); *Hedraea* Turner, 1926; *Hyphorma* Walker, 1865; and *Hyphormides* Hering, 1931 [6,7,8,9,10], exhibit distinct elongated labial palpus. Among these, the labial palpus of *Scopelodes* is particularly notable for the presence of a conspicuous hair tuft at the apex. Within the Oriental region, *Hyphormides* and *Scopelodes* form a group with *Hyphorma*, as they share a distinctive elongation of the labial palpi (Holloway, 1986) [11]. Solovyev & Witt (2009) [12] described a new genus, *Tanvia Solovyev & Witt*, 2009, closely allied to *Scopelodes* with elongated labial palpus and an apical hair tuft. Later, Solovyev & Giusti (2017) [13] further confirmed that members of *Scopelodes* are related to those in the genera *Tanvia* and *Hyphorma* by their appearance.

More recently, Liang et al. (2024) [4] provided molecular evidence demonstrating that genera from the Palaearctic and Indomalayan regions, including *Scopelodes*, *Tanvia*, *Monema*, and *Hyphorma*, form a well-supported lineage within the *Parasa*-clade, corroborating earlier morphological studies. Building upon these findings, the present study will select representative taxa from these genera for a detailed comparative analysis with the newly described genus. Specifically, it aims to (1) formally describe the new genus and species using comprehensive morphological and molecular data, and (2) investigate its phylogenetic position within Limacodidae, with a focus on its affinities to morphologically similar genera, such as *Scopelodes* and *Tanvia*.

## 2. Material and Methods

### 2.1. Sampling

The specimens examined in this study were collected and used for DNA extraction. Initially, three legs from one side of each individual were removed, preserved in 100% alcohol, and stored at −20 °C for further analysis. For phylogenetic analysis, we selected two Zygaenidae sequences, *Eterusia aedea* (Linnaeus, 1763) and *Histia flabellicornis* (Fabricius, 1775), from BOLD/NCBI as outgroups, based on the close phylogenetic relationship between Limacodidae and Zygaenidae (Bian et al. (2020) [14]). To ascertain the phylogenetic placement of the new genus within Limacodidae, we incorporated several published sequences alongside newly sequenced data, including those of the new species. These sequences represent genera *Tanvia*, *Scopelodes*, *Hyphorma*, and *Monema*, all of which belong to the *Parasa*-clade as defined by Liang et al. (2024) [4]. Morphologically, the new genus shares features with these genera, such as extended labial palpi and comparable wing venations. In addition, we also included the genus *Mahanta* Moore, 1879 [15] as a comparative genus. Although it lacks a distinctly elongated labial palpus, it clusters with *Scopelodes* or *Monema* in the phylogenetic tree. The collecting locality, BOLD sample IDs, and GenBank accession numbers are listed in Table 1. All sequences of the new species and the newly sequenced data presented in this paper have been deposited in GenBank under Accession Numbers PQ561650–PQ561656 (Table 1).

### 2.2. Morphological Study

Traditional insect taxonomic methods were used for the morphological study. Specimens were collected at night using a high-pressure 220V/450W mercury lamp supplemented with an 8V/5W DC blacklight. Genitalia preparations followed the method described by Kononenko & Han (2007) [16]. Abdomens were detached with fine tweezers and soaked in a 10% KOH solution for 12–24 h. After defatting, genitalia were separated in 75% ethanol for further cleaning, dehydrated in 100% ethanol, stained, and then fixed in xylene for observation and photography. To examine the vesica, the phallus was removed, and the vesica everted using a 1 mL syringe to expose internal structures such as cornuti. Permanent slides were mounted with Canada balsam.

Adult specimens were photographed with Nikon D700 (Nikon Corporation, Tokyo, Japan) and Canon M6 Mark II (Canon Inc., Tokyo, Japan) cameras to document observable morphological traits, including body color, forewing length, wingspan, and wing patterns. Specimen measurements were taken with a ruler; forewing length was measured from apex to base, and wingspan from apex to apex. Observations and measurements of genital structures on slides were conducted under a microscope. Genitalia slide photographs were taken with an AOSVI Hk-830 microscope (Shenzhen Aoswei Optical Instruments Co., Ltd., Shenzhen, China), with images refined using Helicon Focus 7 (Helicon Soft Ltd., Kharkiv, Ukraine) and Adobe Photoshop 2020 (Adobe Inc., San Jose, CA, USA) for enhanced clarity. The collecting map was constructed using the QGIS v3.40 software.

The terminology for adult and genital structures follows Epstein (1996) [17] and Kristensen (2003) [18]. All type materials of the new species are deposited in the collection of the Northeast Forestry University (NEFU), Harbin, China.

### 2.3. DNA Extraction and PCR

Standard DNA extraction and amplification methods were performed. Genomic DNA was extracted from three legs of each specimen using the TaKaRa MiniBEST Universal Genomic DNA Extraction Kit Ver.5.0 (Takara Bio Inc., Shiga, Japan), following overnight incubation at 56 °C and according to the kit’s protocol. Polymerase chain reactions (PCRs) were conducted on a PCR Thermal Cycler (Hangzhou LongGene Scientific Instruments Co., Ltd., Hangzhou, China) using the primers LCO1490 and HCO2198 (Folmer et al. (1994) [19]) to amplify a 658 bp fragment of the mitochondrial cytochrome c oxidase I (COI) gene, as listed in Table 2.

The total reaction volume was 25 μL, consisting of 0.5 μL template DNA, 10 μL ddH2O, 12.5 μL 2× Rapid Taq Master Mix (Vazyme Biotech Co., Ltd., Nanjing, China), and 1 μL each of forward and reverse primers (synthesized by Sangon Biotech from Changchun, China). The PCR program included an initial denaturation at 95 °C for 3 min; 35 cycles of 95 °C for 30 s, 55 °C for 30 s, and 72 °C for 1 min; with a final extension at 72 °C for 5 min.

### 2.4. Molecular Phylogenetic Analysis

All amplicons were sequenced using an ABI 3730XL automated sequencer. The raw sequences were initially manually corrected with Chromas v2.6.6 software (Technelysium Pty Ltd., Brisbane, Australia), followed by assembly of the bidirectional sequences in Geneious v9.0.2 (https://www.geneious.com, accessed on 27 October 2024). Phylogenetic analysis was conducted with sequences obtained in this study along with additional Limacodidae sequences downloaded from the GenBank database (https://www.ncbi.nlm.nih.gov/nucleotide, accessed on 23 July 2024) and BOLD database (Barcode of Life Data Systems, Guelph, Canada, https://v4.boldsystems.org/, accessed on 23 July 2024). PhyloSuite 1.2.3 (Zhang et al. (2020) [20]; Xiang et al. (2023) [21]) was used for the analysis, and 20 sequences were aligned with MAFFT v7.505 (Katoh and Standley (2013) [22]) using ‘--auto’ strategy and normal alignment mode. Ambiguously aligned fragments of 1 alignment was removed using Gblocks 0.91b (Talavera and Castresana (2007) [23]) with the following parameter settings: minimum number of sequences for a conserved/flank position (11/11), maximum number of contiguous non-conserved positions (8), minimum length of a block (10), allowed gap positions (with half).

ModelFinder v2.2.0 (Kalyaanamoorthy et al. (2017) [24]) was used to select the best-fit model using the BIC criterion. Phylogenetic analyses were performed using the Bayesian inference (BI) approach. Bayesian posterior probabilities were estimated in MrBayes v3.2.7a (Ronquist et al. (2012) [25]), with the best-fit model according to ModelFinder’s BIC recommendation: GTR+G+F. Sampling was conducted every 1000 generations. We conducted Bayesian inference using MCMC with four chains (one cold and three hot) for each run. The analysis was performed for 5 million generations in two independent runs to ensure convergence and robustness of the results, discarding the first 25% of sampled trees as burn-in. Tree nodes with BI posterior probabilities (PPs) > 0.95 were considered well-supported. The final phylogenetic tree was visualized using ChiPlot (Xie et al. (2023) [26]) (https://www.chiplot.online/index.html, accessed on 20 October 2024) and edited with Adobe Illustrator 2023.

Simultaneously, we aligned sequences using Clustal W in MEGA 11 (Tamura et al. (2021) [27]) and checked for stop codons after translating them to amino acid sequences. Uncorrected pairwise genetic distances of the COI gene were calculated among all individuals of the new species and the type species of each related genus. The analysis, comprising 8 nucleotide sequences, was conducted using the Kimura 2-parameter model (Kimura (1980) [28]), with codon positions set to 1st+2nd+3rd+Noncoding. All ambiguous positions were removed for each sequence pair (pairwise deletion option), yielding 658 positions in the final dataset. Evolutionary analyses were conducted in MEGA 11.

## 3. Result

### 3.1. Taxonomic Treatment

*Guium* Wu & Han, gen. nov.
Zoobank: urn:lsid:zoobank.org:act:C66396B0-701A-4CBD-BA49-CEBF3974A9BD
Type species: *Guium nebulum* Wu & Han, sp. nov.
Gender: neutral
Chinese name: 桂刺蛾属

**Figure 1:**
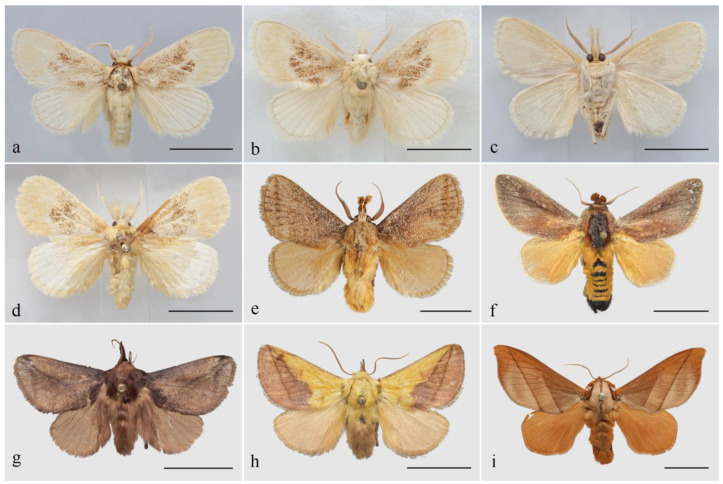
Male adults. (**a**) *Guium nebulum* sp. nov., holotype (Guangxi, China); (**b**,**c**) ditto, paratype, dorsal and ventral side (Guangxi, China); (**d**) ditto, paratype (Jiangxi, China); (**e**) *Tanvia zolotuhini* Witt & Solovyev, 2009 (Yunnan, China); (**f**) *Scopelodes unicolor* Westwood, 1841 (Borneo); (**g**) *Hyphorma minax* Walker, 1865 (Chongqing, China); (**h**) *Monema flavescens* Walker, 1855 (Heilongjiang, China); (**i**) *Mahanta tanyae* Solovyev, 2005 (Guizhou, China). Scale bars: 10 mm, all in NEFU.

**Diagnosis:** The genus *Guium* (Figure 1a–d) shares the characteristic of a noticeably extended labial palpus with related genera such as *Tanvia*, *Scopelodes*, *Hyphorma*, *Monema*, and *Mahanta* (Figure 1e–i), but exhibits several distinct features that clearly differentiate it from them. *Guium* is characterized by its pale-yellow coloration and a large, diffuse ochre blotch on the forewing. In contrast, these related genera have a darker overall coloration, with predominant shades of ochre, brown, or dark brown. In the male antennae, *Guium* is similar to those of *Tanvia*, with bipectinate from base to apex, gradually narrowing; *Scopelodes* and *Hyphorma* are broadly bipectinate in the basal 1/2 to 2/3; those of *Monema* are filiform; and in *Mahanta*, only short rami are present.

**Etymology:** The genus name is derived from “Gui”, an abbreviation for Guangxi Autonomous Region, China, the type locality of the new genus.

**Figure 2:**
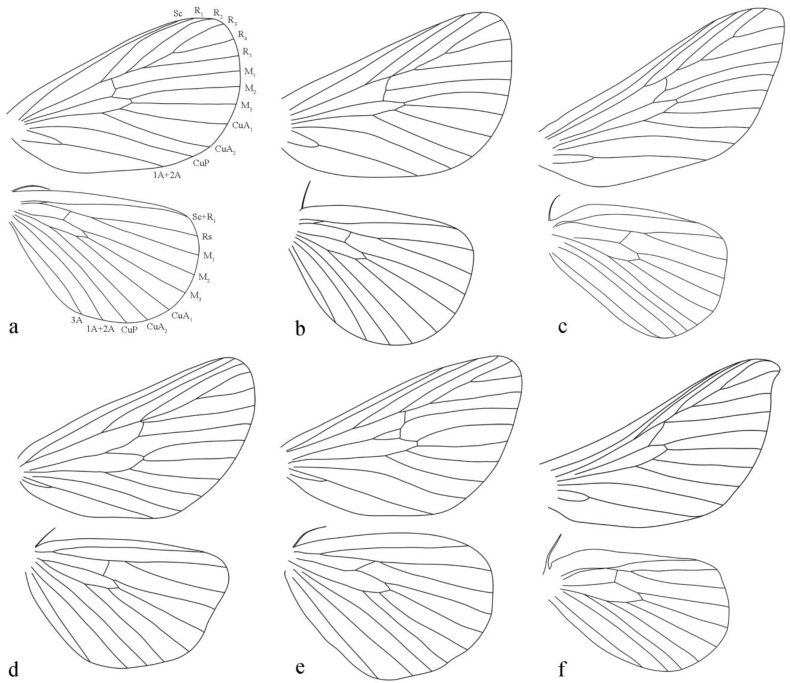
Wing venations. (**a**) *Guium nebulum* sp. nov.; (**b**) *Tanvia zolotuhini* Witt & Solovyev, 2009; (**c**) *Scopelodes unicolor* Westwood, 1841; (**d**) *Hyphorma minax* Walker, 1865; (**e**) *Monema flavescens* Walker, 1855; (**f**) *Mahanta tanyae* Solovyev, 2005.

The forewing venation of *Guium* (Figure 2a) is distinct: vein R_1_ has a pronounced outward curve and approaches Sc proximally, with the upper angle of the discal cell nearly a right angle and the lower angle extending noticeably outward. In case of the related genera (Figure 2b–f), R_1_ is straight, and does not approach Sc, while the upper angle forms a sharp point, and the lower angle is only slightly outward, level with, or shorter than the upper angle. Additionally, *Guium* resembles *Scopelodes* (Figure 2c) and *Mahanta* (Figure 2f) in having R_2_ branch after the upper angle of the discal cell and share a stem with R_3+4+5_. By contrast, in *Tanvia* (Figure 2b) and *Hyphorma* (Figure 2d), R_2_ branches near the upper angle, and in *Monema* (Figure 2e), R_2_ originates before this angle.

**Figure 3:**
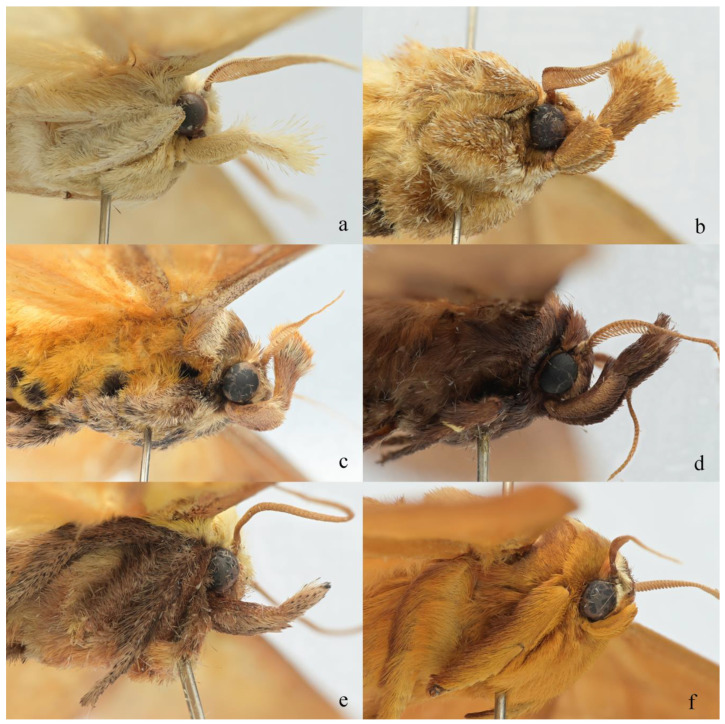
Labial palpi. (**a**) *Guium nebulum* sp. nov.; (**b**) *Tanvia zolotuhini* Witt & Solovyev, 2009; (**c**) *Scopelodes unicolor* Westwood, 1841; (**d**) *Hyphorma minax* Walker, 1865; (**e**) *Monema flavescens* Walker, 1855; (**f**) *Mahanta tanyae* Solovyev, 2005.

The labial palpus of *Guium* (Figure 3a) resembles that of *Tanvia* (Figure 3b) and *Scopelodes* (Figure 3c) in being elongated with a tuft of scales at the end of the third segment. However, the tuft in *Guium* is loosely arranged and not distinctly globular. Genera *Hyphorma*, and *Monema*, (Figure 3d,e) also have elongated palpi, but the third segment is covered with short scales and lacks a tufted appearance. The labial palpus in *Mahanta* (Figure 3f) is short.

**Figure 4:**
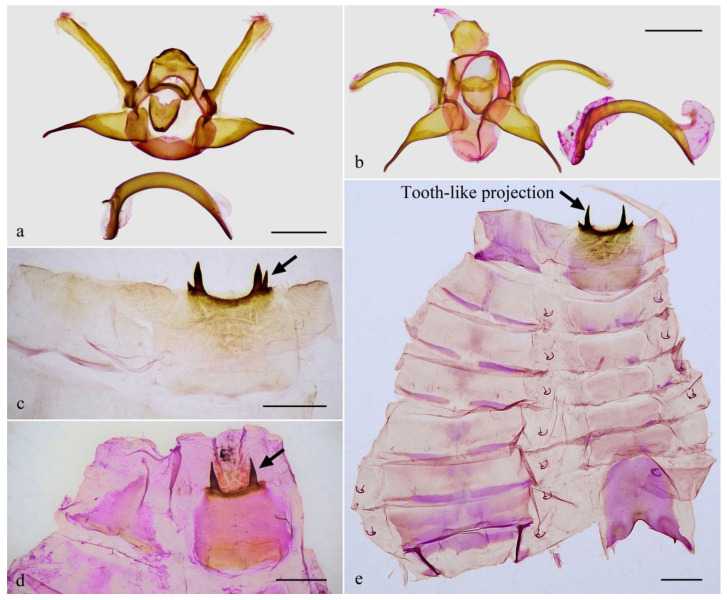
Male genitalia (**a**,**b**), abdominal segment VIII (**c**,**d**), and abdomen (**e**) of *Guium nebulum* sp. nov. (**a**,**c**) holotype, slide WuJ-740-1 (Guangxi, China); (**b**,**d**) paratype, slide WuJ-1137-1 (Jiangxi, China); (**e**) paratype, slide WuJ-709-1 (Guangxi, China). In (**c**–**e**) the dorsum is shown on the left and the ventrum on the right, with the tooth-like projections indicated by arrows. Scale bars: 1 mm, all in NEFU.

**Figure 5:**
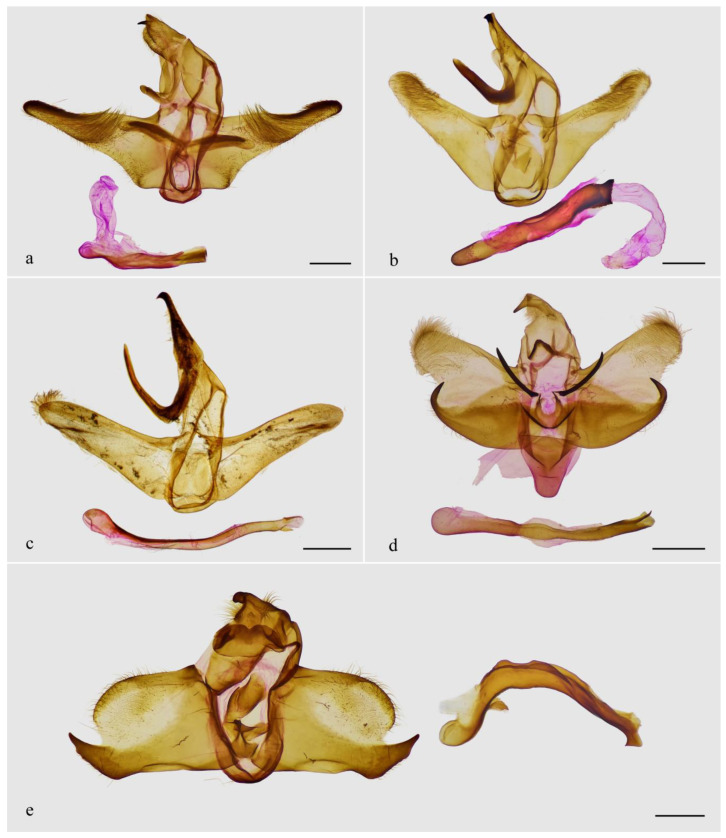
Male genitalia of related genera. (**a**) *Tanvia zolotuhini* Witt & Solovyev, 2009, slide WuJ-1037-1; (**b**) *Scopelodes unicolor* Westwood, 1841, slide WuJ-1142-1; (**c**) *Hyphorma minax* Walker, 1865, slide WuJ-116-1; (**d**) *Monema flavescens* Walker, 1855, slide WuJ-976-1; (**e**) *Mahanta tanyae* Solovyev, 2005, slide WuJ-1043-1. Scale bars: 1 mm, all in NEFU.

The male genitalia of *Guium* (Figure 4a,b) shows distinctive modifications: the uncus is broadened without an apical spine; the gnathos is wide and band-shaped; and the valva is divided into upper and lower lobes. Additionally, the eighth abdominal segment (Figure 4c–e) bears a pair of robust, sclerotized, tooth-like projections, each with one or more small spines at the base. These unique features in the male genitalia and abdomen are absent in related genera with elongated labial palpus mentioned herein, which exhibit the typical Limacodidae structure (Figure 5a–d). The male genitalia of *Mahanta* species (Figure 5e) share some similarities with those of the new genus, including the blunt uncus, the bifurcated valva, and a strongly curved phallus. However, the two genera can be distinguished by their general appearance.

**Description:** Male antennae bipectinate from base to apex, gradually narrowing. Labial palpus grayish-brown, extending prominently; first segment very short, second segment thicker, third segment tapering, ending with a tuft of long, pale yellow scales. Thorax dorsally grayish-white with a faint brownish tinge. Forewing broad with a pale-yellow base color, featuring a large, ochreous misty patch. Hindwing grayish-white. Forewing venation: vein R_1_ strongly curved distally, closely approaching vein Sc; R_2_ shares a stalk with veins R_3_ + R_4_, and R_5_; R_5_ originates after R_2_; M_3_ emerges from the lower angle of discal cell. Hindwing: Rs and M_1_ veins share a common stalk. Legs grayish-white; tibial spurs in formula 0-2-4. Sternite VIII bears a pair of hardened, tooth-like projections, each with one or more small spines at base.

**Male genitalia.** Highly modified compared to the typical male genitalia structure of Limacodidae, such as those of the related genera *Tanvia* and *Scopelodes*. Uncus wide, without apical spine; gnathos broad, band-shaped; tegumen broad. Valva divided into upper and lower lobes: upper lobe narrow, elongated; lower lobe sclerotized, with the basal half expanded. Juxta shield-like. Saccus indistinct. Phallus tubular, strongly sclerotized, down curved into a bow shape, with oblique slits near the caecum and apex.

**Female**. Unknown.

**Remark**: This genus is known to be a monotypic genus and is distributed in the Guangxi Autonomous Region and Jiangxi Province, China.

*Guium nebulum* Wu & Han, sp. nov.
Zoobank: urn:lsid:zoobank.org:act:7E92DB49-1E2A-465B-89A0-2B1D3BE3C1D4
Figure 1a–d, Figure 2a, Figure 3a and Figure 4a–e
Chinese name. 桂刺蛾

**HOLOTYPE**: ♂, Guangxi Yinzhulaoshan Ziyuan Fir National Nature Reserve, Ziyuan County, Guilin City, Guangxi Autonomous Region, China, 26°16′15″ N, 110°35′44″ E, 2021-VII-15–18, Jun-Jie Fan, Biao Gao *leg*., genit. prep. WuJ-740-1 [NEFU]. **PARATYPES**: 4 ♂, same data as for holotype, genit. prep. WuJ-709-1, WuJ-739-1 [NEFU]. 1 ♂, Dazhangshan Township, Wuyuan County, Shangrao City, Jiangxi Province, China, 29°27′07″ N, 117°44′02″ E, 2024-VI-30, Kai Wu, Xin-Yu Cheng *leg*., genit. prep. WuJ-1137-1 [NEFU].

**Diagnosis:** The new species is similar to members of *Tanvia* and *Scopelodes*, in having a tuft of long scales at the end of the third labial palpus segment, but easily distinguished by appearance, male genitalia, and sternite VIII, as described for the genus.

**Description: Male.** Forewing length 14–15 mm, wingspan 30–34 mm (*N* = 6). Head pale grayish-white; antennae bipectinate till to tip. Labial palpus grayish-brown, extending prominently; first segment very short, second segment thicker, third segment tapering, approximately 4/5 the length of the second, ending with a tuft of long, pale yellow, loosely arranged scales. Thorax dorsally grayish-white with a faint brownish tinge. Forewing broad, with a pale-yellow base color, featuring a large, ochreous misty patch extending from approximately two-thirds along costa to halfway down the inner margin, scattered with dark brown scales; outer region without distinct markings. Terminal line distinct, thin, brown; fringes grayish-white. Hindwing grayish-white, with a faint brownish tinge along anal margin; fringes as in the forewing. Abdomen grayish-white with a faint brown; sternite VIII bears a pair of hardened, tooth-like projections, each with one or more small spines at base.

**Male genitalia.** Uncus wide, without apical spine; gnathos broad, band-shaped; tegumen broad. Valva divided into upper and lower lobes: upper lobe narrow, elongated, with a prominent, strongly sclerotized basal “pedestal”, with its upper edge weakly sclerotized, lower edge membranous; lower lobe sclerotized, with the basal half expanded, tapering sharply at midsection, ending in a strongly sclerotized, blunt rod-like process. Juxta shield-like, widening at top, narrowing below, edges strongly sclerotized with a notch at the mid-dorsal margin. Saccus indistinct. Phallus tubular, strongly sclerotized, down curved into a bow shape; caecum and apex each with long oblique slits, approximately 1/4 and 1/3 of the total phallus length, respectively.

**Distribution**: China (Guangxi, Jiangxi).

**Bionomics**: The new species was collected from montane regions in Guangxi Autonomous Region and Jiangxi Province, China. Specimens were captured in middle July in Guangxi and late June in Jiangxi, indicating adult activity during summer in these regions. The collection sites are characterized by mixed forest ecosystems, suggesting that the species may prefer forested habitats at low to mid elevations.

**Etymology**: The specific epithet is deriving from the Latin word nebula, meaning “mist” or “fog”, in reference to the ochre mist-like patch on the forewings of the new species.

### 3.2. Molecular Phylogenetic Analysis

**Figure 6:**
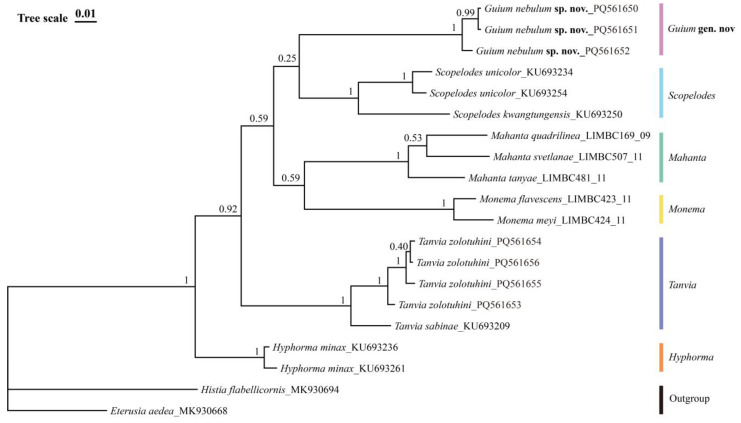
Bayesian inference based on the DNA barcode sequences (COI) and rooted on two Zygaenidae species as outgroups. Nodal numbers are BI posterior probability values.

Bayesian phylogenetic analysis showed good convergence, indicated by an average standard deviation of split frequencies close to 0 (0.004307) and potential scale reduction factors (PSRF) equal to 1 (maximum = 1.001). The topology generated by Bayesian inference is consistent with the results of Liang et al. (2024) [4], which used 13 mitochondrial markers. The new genus in this study formed an independent clade, supporting the monophyly of the *Guium* species (Figure 6).

In the molecular analysis, interspecific distance values in Lepidoptera are generally greater than 3% (Hebert et al. 2003 [29]). In this study, the K2P distances of COI between *G. nebulum* sp. nov. and the type species of each related genus ranged from 9.7% to 12.4%, while the maximum intraspecific distance for *G. nebulum* is only 1.0% (Table 3).

Morphologically, *Guium* species share certain traits with neighboring genera, such as extended labial palpus and similar wing venation, likely indicating a shared evolutionary history. However, molecular analyses reveal phylogenetic divergence between *Guium* gen. nov. and these related genera. Bayesian inference indicate that *Guium* gen. nov. forms a distinct clade. Additionally, unique bifurcated valva and the highly modified sternite VIII in the male genitalia demonstrate marked evolutionary differentiation. These morphological differences may reflect functional adaptations, while molecular data indicate deeper phylogenetic distinctions from related genera.

## 4. Discussion

**Figure 7:**
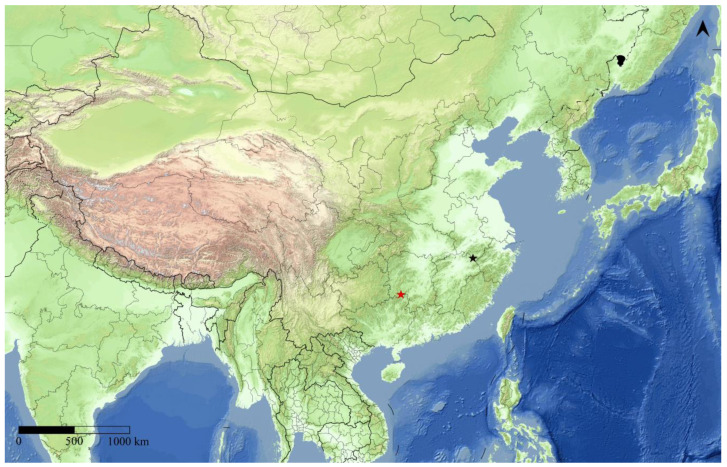
Two collecting sites of *Guium nebulum* sp. nov. The red star indicates the type locality.

In this study, we conducted an integrated analysis of molecular data and morphological characteristics, providing evidence for the establishment of the new genus. Our molecular and morphological data support the validity of *Guium* gen. nov. Although this genus currently includes only the type species, *G. nebulum* sp. nov., found in Guangxi and Jiangxi (Figure 7), China, it may potentially have a broader distribution. Given similar ecological conditions in neighboring regions, such as Hunan and Fujian provinces, and in parts of Southeast Asia with comparable habitats, future surveys may uncover additional species within this genus or expand its known distribution.

Among the three specimens, the two individuals from Guangxi showed no genetic divergence (0%), while the genetic distance between the Guangxi and Jiangxi individuals was 1.0%. Minor genetic divergence within the species may reflect population-level differences; ecological or climatic differences between Guangxi and Jiangxi might be affected by such divergence. In addition to these minor genetic variations, a slight difference in the male genitalia was observed: in the Jiangxi specimen, the upper lobe of the valva is curved, with small spines around the terminal tooth-like projection on sternite VIII, whereas the Guangxi specimen has a relatively straight upper lobe of the valva and more developed spines around the terminal projection of sternite VIII. We hypothesize that these differences might result from localized ecological or genetic factors, but further sampling and analysis across a broader geographic range are required to clarify the reasons behind their occurrence.

Due to the limited molecular marker used in this study (only a 658 bp COI sequence), we were able to confirm the monophyly of the new genus but could not fully resolve its phylogenetic relationships with closely related genera. Future studies should aim to incorporate additional genomic data, particularly large-scale nuclear markers, to clarify the phylogenetic position of *Guium* gen. nov. and its relatives, as well as to provide insights into their evolutionary history. The lack of information on juvenile stages and adult females also limits our ability to compare non-adult stages with closely related genera. Therefore, rearing larvae and documenting life histories are important, as these efforts will not only enhance our understanding of the group’s biology but also help obtain missing female specimens for further taxonomic and phylogenetic analyses.

## Figures and Tables

**Table 1:** Sampling information and BOLD SampleID/GenBank accession numbers of Limacodidae and outgroups used in this study.

Taxon	Voucher No.	Locality	GenBank No.	BOLD ID	Remark
*Guium nebulum* sp. nov.	WJ22-038 (PT)	Guangxi, China	PQ561650	–	this study
*G. nebulum* sp. nov.	WJ22-039 (PT)	Guangxi, China	PQ561651	–	this study
*G. nebulum* sp. nov.	WJ24-672 (PT)	Jiangxi, China	PQ561652	–	this study
*Hyphorma minax*	WITT-LIMAC-240	Hubei, China	KU693236	LIMBC605-11	
*H. minax*	AS-LIMAC 348	Louangphrabang, Laos	KU693261	LIMBC886-11	
*Monema flavescens*	WITT-LIMAC-058	Shaanxi, China	–	LIMBC423-11	
*M. meyi*	WITT-LIMAC-059	Guangxi, China	–	LIMBC424-11	
*Mahanta quadrilinea*	AS-LIMAC 174	Trongsa, Bhutan	–	LIMBC169-09	
* M. tanyae *	WITT-LIMAC-116	Shaanxi, China	–	LIMBC481-11	
* M. svetlanae *	WITT-LIMAC-142	Yunnan, China	–	LIMBC507-11	
*Scopelodes unicolor*	WITT-LIMAC-342	Sumatera Barat, Indonesia	KU693254	LIMBC712-11	
*S. unicolor*	AS-LIMAC 279	Sabah, Malaysia	KU693234	LIMBC317-10	
*S. kwangtungensis*	WITT-LIMAC-364	Mechi, Nepal	KU693250	LIMBC734-11	
*Tanvia sabinae*	WITT-LIMAC-368	Chiang Mai, Thailand	KU693209	LIMBC763-11	
*T. zolotuhini*	WJ19-039	Yunnan, China	PQ561653	–	this study
*T. zolotuhini*	WJ19-040	Yunnan, China	PQ561654	–	this study
*T. zolotuhini*	WJ19-041	Yunnan, China	PQ561655	–	this study
*T. zolotuhini*	WJ19-042	Yunnan, China	PQ561656	–	this study
*Eterusia aedea*	ChChEt#tri#003	Yunnan, China	MK930668	ZYGMO614-13	out group
*Histia flabellicornis*	ChChHi#flacom003	Sagaing, Myanmar	MK930694	ZYGMO620-13	out group

“PT” means paratype; “–” means no available sequences from GenBank or BOLD.

**Table 2:** PCR primers used to sequence COI genes of limacodid species in this study.

Primer	Sequence	Reference
LCO1490	GGTCAACAAATCATAAAGATATTGG	Folmer et al. (1994) [19]
HCO2198	TAAACTTCAGGGTGACAAAAAATCA	Folmer et al. (1994) [19]

**Table 3:** Pairwise K2P distances of COI sequences for new species and the type species of each related genus used in this study.

Species Code	Voucher No.	Taxon	1	2	3	4	5	6	7
1	WJ22-038 (PT)	* Guium nebulum * sp. nov.							
2	WJ22-039 (PT)	*G. nebulum* sp. nov.	0.000						
3	WJ24-672 (PT)	* G. nebulum * sp. nov.	0.010	0.010					
4	WITT-LIMAC-342	* Scopelodes unicolor *	0.101	0.101	0.097				
5	WJ19-040	*Tanvia zolotuhini*	0.113	0.113	0.123	0.114			
6	WITT-LIMAC-058	* Monema flavescens *	0.123	0.123	0.124	0.108	0.127		
7	AS-LIMAC 174	*Mahanta quadrilinea*	0.107	0.107	0.110	0.104	0.138	0.119	
8	WITT-LIMAC-240	* Hyphorma minax *	0.101	0.101	0.101	0.092	0.101	0.101	0.110

“PT” means paratype.

## Data Availability

Data are contained within the article.
